# Mitochondrial Dynamics: A Key Role in Neurodegeneration and a Potential Target for Neurodegenerative Disease

**DOI:** 10.3389/fnins.2021.654785

**Published:** 2021-04-12

**Authors:** Danying Yang, Jun Ying, Xifeng Wang, Tiancheng Zhao, Sungtae Yoon, Yang Fang, Qingcui Zheng, Xing Liu, Wen Yu, Fuzhou Hua

**Affiliations:** ^1^Department of Anesthesiology, The Second Affiliated Hospital of Nanchang University, Nanchang, China; ^2^Key Laboratory of Anesthesiology of Jiangxi Province, Nanchang, China; ^3^Department of Anesthesiology, The First Affiliated Hospital of Nanchang University, Nanchang, China; ^4^Mailman School of Public Health, Columbia University, New York, NY, United States; ^5^Helping Minds International Charitable Foundation, New York, NY, United States

**Keywords:** mitochondrial dynamics, mitochondrial fusion and fission, mitophagy, mitochondrial transport, neurodegeneration

## Abstract

In neurodegenerative diseases, neurodegeneration has been related to several mitochondrial dynamics imbalances such as excessive fragmentation of mitochondria, impaired mitophagy, and blocked mitochondria mitochondrial transport in axons. Mitochondria are dynamic organelles, and essential for energy conversion, neuron survival, and cell death. As mitochondrial dynamics have a significant influence on homeostasis, in this review, we mainly discuss the role of mitochondrial dynamics in several neurodegenerative diseases. There is evidence that several mitochondrial dynamics-associated proteins, as well as related pathways, have roles in the pathological process of neurodegenerative diseases with an impact on mitochondrial functions and metabolism. However, specific pathological mechanisms need to be better understood in order to propose new therapeutic strategies targeting mitochondrial dynamics that have shown promise in recent studies.

## Introduction

Neurodegenerative diseases are a kind of incurable and devastating neurological disorders, in these diseases, neurons gradually lose their function and ultimately cause death. The increased longevity of people’s lives has contributed to a rising incidence rate of neurodegenerative disorders associated with aging over the past 50 years. Due to the complexity of brain function, disease-modifying treatments of neurodegenerative diseases remain elusive despite concerted attempts to rescue brain energy, counter neuroinflammation or neurotoxic protein.

Mitochondria undergoing coordinated cycles of fission and fusion, the balance of fission/fusion events is referred to as the “mitochondrial dynamic.” Energy for neurons is provided by mitochondria, who generate adenosine triphosphate (ATP) via oxidative phosphorylation. Excessive fragmentation or dysfunction of mitochondria may have an impact on the ATP biogenesis and lead to mitochondrial dysfunction, which can be disastrous for the survival viability of neurons. In this review, three mitochondrial dynamic related events are discussed: the fusion/fission event, movement within the neuronal axon, and disintegration of mitochondria (mitophagy).

Due to the essential nature of mitochondrial functions, excessive mitochondrial fission leads to brain dysfunction. Characteristic hallmarks in the brains of patients with neurodegenerative diseases such as the accumulation of a-synuclein, hyperphosphorylated tau, and amyloid beta (Aβ) plaques, have been proven to be related to the activity of mitochondrial fission proteins. We focus on the impaired mitophagy pathway represented by PINK/parkin defects in neurodegenerative diseases, the important role of the ubiquitin protease system (UPS), and the potential connection between mitochondrial dynamics and the pathological process of neurodegenerative diseases. In addition to its role in quality control, deficiencies in PINK/Parkin may also lead to neuroinflammation, thus exacerbating neurodegenerative disease progression. The mechanisms of mitochondrial dynamics cause an imbalance in several neurodegenerative diseases and allow us to have a better understanding of the pathogenesis, while altered peripheral blood mitochondrial dynamics provide a potential diagnostic approach for Alzheimer’s disease.

Finally, we propose possible future research directions for mitochondrial dynamics and consider the challenge of translating promising therapeutic targets toward the dual goals of symptom relief and disease modification in Neurodegenerative diseases.

## Mitochondrial Fission and Fusion

Mitochondrial fission and fusion play critical roles in creating new mitochondria and removing damaged mitochondria. In mammalian cells, fission/fusion events are mainly mediated by several large dynamin-related GTPase proteins, including conserved dynamin-related GTPase (Drp1), conserved dynamin-related GTPase mitofusion (Mfn), and optic dominant atrophy 1 (Opa1). In addition to the GTPase activity of these proteins, fission/fusion processes are regulated by multiple mechanisms, and this review provides a short description of the mechanisms that control mitochondrial fission and fusion.

**FIGURE 1:**
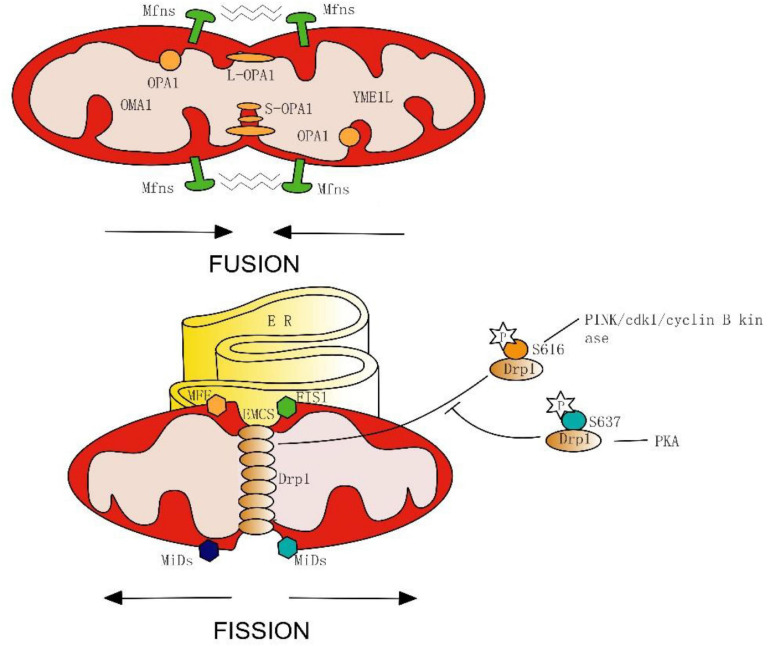
Simplified model of mitochondrial fusion and fission in mammals. Fusion is mediated on the OMM by the Mfns, and on the IMM by Long forms (L-OPA1) or short forms (S-OPA1) of OPA1, which spliced from OMA1 and YME1L. Fission begins with endoplasmic reticulum (ER) contacts with OMM at the ER-mitochondria contact sites (EMCS). Next, mitochondrial-bound proteins (FIS1, MFF, and MiDs) aid in the oligomeric forms of Drp1, and induce eventually fission. Phosphorylation of Drp1 serine 616 promotes its oligomerization, whereas phosphorylation of Drp1 serine 637 inhibits mitochondrial fission.

In the initial stage of mitochondrial fission, the endoplasmic reticulum (ER) makes contact with the outer mitochondrial membrane (OMM) at the ER-mitochondria contact sites (EMCS) and causes mitochondrial constrictions (Friedman et al., 2011) (Figure 1). Some mitochondrial-bound proteins work as “membrane-anchored adaptors,” including FIS1, MFF, and MiDs (Otera et al., 2010; Zhao et al., 2011), which aid the oligomeric forms of Drp1 recruited to EMCS. Then, Mitochondria-bound Drp1 form a ring-like structure, enhancing the pre-existing mitochondrial constriction neck and eventually inducing fission. On-going investigations in this area have uncovered the important roles of EMCS in mitochondrial dynamics and related diseases. EMCS is necessary for correct mitochondrial function, by connecting mitochondrial stability to intracellular calcium handling (Lynes and Simmen, 2011; Rowland and Voeltz, 2012). A new study has proposed a mechanism of nucleoid called active transportation, which focuses on the interaction of nucleoids, MICOS, Miro1, and KFI5B, aiming to implement the coordination of the nucleoid segregation and transportation during mitochondrial fusion and division (Qin et al., 2020). EMCS is regarded as the essential platforming in this mechanism. ER-mitochondrial contact is an independent and conservative feature, and Drp1 also plays a vital role in mitochondrial division.

Post-translational modification is an important mechanism for GTPase activation, phosphorylation of Drp1 is the most widely discussed example. During mitosis, serine 616 on the GED of Drp1 is phosphorylated by cdk1/cyclin B kinase, stimulating its oligomerization and results in mitochondrial fission (Taguchi et al., 2007; Kashatus et al., 2015). Interestingly, new seminal work has proposed that the PINK1 phosphorylating Drp1 on S616 is feasible, by which the mitochondrion can be split into the damaged part and the healthy part by promoting the fission process. After the fission process, the PINK1/parkin-mediated mitophagy will further degrade the damaged mitochondria. Thus, PINK1 may have a role in both fission and mitophagy during the mitochondrial quality control (Han H. et al., 2020). Furthermore, the PKA is capable of phosphorylating the serine residue (637) as well, which inhibits fission and protects mitochondria from autophagosomal degradation during nutrient deprivation (Cereghetti et al., 2008; Gomes et al., 2011; Chou et al., 2012). From these results, we can conclude that the physiological properties of Drp1 are inseparable from its phosphorylation site.

Mitochondrial fusion includes two inseparable progressions. In mammals, OMM fusion is ensured by Mfn1 and Mnf2– dynamin-like GTPases with conserved catalytic domains and the fusion of the inter mitochondrial membrane (IMM) is mediated by OPA1 and mitochondria-specific cardiolipin (CL) (Kameoka et al., 2018).

Similarly, the GTPase activity of Mfn1 is regulated by ubiquitination and acetylation, Mfn2 can also be ubiquitinated (Ziviani and Whitworth, 2010). Long forms (L-OPA1) or short forms (S-OPA1) of OPA1 can be generated through the mRNA splicing of IM peptidases like OMA1 and YME1L (Anand et al., 2014). Different forms of OPA1 may manifest distinct subcellular localizations and functions (Ishihara et al., 2006; Anand et al., 2014). Thus, the properties of the GTPase are not static, and their function can be changed or even turned to the opposite through modification methods such as pre-translation modifications or splice. This means that we can not only inhibit or promote GTPase activity but also “convert” it.

Notably, IMM fusion occurs downstream of OMM fusion, and OPA1-mediated IMM fusion depends on Mfn2 but not Mfn1 (Cipolat et al., 2004; Ishihara et al., 2004), Mfn1 and Mfn2 may play different roles in the fusion process of mitochondria. However, the focus of past research has been Mfn2, and thus the potential interaction of Mfn1 with OPA1 remains unclear. Together, mitochondrial fission or fusion is a coherent dynamic process involving multiple mechanisms, which implies that once a certain link changes, it will have a broad impact on mitochondrial dynamics, even causing neuronal dysfunction. This requires us to pay closer attention to the chain reaction and downstream effects in the treatment of mitochondrial dynamics.

The role of phospholipids like cardiolipin (CL) and phosphatidic acid (PA) in remodeling and regulating mitochondrial dynamics has been revealed in past research (Kameoka et al., 2018). PA can be transferred directly from the ER to the mitochondria as a saturated lipid, then it stimulates OPA1 assembly and GTPase activity by converting to CL at the OMM, which subsequently promotes OMM fusion. Correspondingly, a small amount of CL can be converted into PA by mitoPLD at OMM (Choi et al., 2006; Huang et al., 2011; Vance, 2015), and accumulation of PA enhances Mfns-dependent OMM fusion. CL binds to Drp1 to enhancing constriction and tubulation of liposome membranes by stimulating oligomerization and GTPase activity of Drp1 (Bustillo-Zabalbeitia et al., 2014; Stepanyants et al., 2015). However, Drp1 binds to PA synthesis via mitoPLD, leading to its oligomerization but inhibiting its GTPase activity, resulting in mitochondrial hyperfusion (Adachi et al., 2016). Thus, PA and CL may play an antagonistic role in mitochondrial fission and fusion regulation.

A recent study shows other substances that affect mitochondrial dynamics include ceramides (C18 and C16), CerS1, ER/mitochondria trafficking, and sphingosine-1-phosphate in mitochondria (Fugio et al., 2020). Other studies have identified that mitochondrial fission process 1 (MTFP1) and MSTO1 (Misato) can modify mitochondrial dynamics, either directly or indirectly (Gal et al., 2017; Morita et al., 2017). However, the mechanisms underlying this process await further elucidation. Due to the complexity and diversity of the mechanism of mitochondrial fusion and fission, we cannot balance mitochondrial dynamics by simply changing the quantity or nature of a certain substance. The elucidation of the influencing factors of mitochondrial fusion and division and its mechanism may help us get closer to the “balance point” of mitochondrial dynamics. Further work to elucidate the molecular mechanisms or signaling pathways that regulate mitochondrial dynamics should help in understanding mitochondrial biocomplexity, metabolism, and dynamics homeostasis, and thereby identify new therapeutic targets in human pathologies.

## Mitophagy

Mitochondria are commonly known as the “powerhouse” of the cell. This is beause mitochondrial quality is important for energy production. In 2005, the scientific community first proposed the term “Mitophagy” to describe a subcellular process involving specific sequestering and degradation of the aged and damaged mitochondria. In normal physiological conditions, mitochondria maintain the quality control process via mitophagy to replenish vital macromolecular precursors for cells and protect against both neuronal cell death and the accumulation of dysfunctional mitochondria simultaneously (Ma et al., 2020). On the contrary, fission is downregulated during starvation-induced autophagy, causing elongation of mitochondrial tubules and thus preventing degradation (Rambold et al., 2011).

Previous studies strongly focus on the partnership between the PINK1[Phosphatase and Tensin homologue (PTEN)-induced kinase 1] and the ubiquitin E3 ligase Parkin. Mutations in this cytoprotective PINK-Parkin pathway are a common cause of Parkinsons disease (Kim et al., 2008; Gaudioso et al., 2019; Yoboue and Valente, 2020). Under cellular or environmental stress, the mitochondrial serine/threonine protein kinase PINK1 accumulates on the OMM for autophosphorylation. It also phosphorylates the ubiquitin molecules on the mitochondrial surface, which contributes to subsequent Parkin activation. Indeed, PINK1 was shown to straightforwardly phosphorylate the ubiquitin-like domain (Ubl) of Parkin, subsequently, Parkin ubiquitinates specific proteins like Mfns and FIS1 (Junqueira et al., 2019). In addition, by cooperating with PINK1 and Parkin, Drp1 is recruited to the OMM, leading to mitochondrial fragmentation and thus promoting mitophagy, thus PINK and Parkin form a particular way for organelle degradation (Yoboue and Valente, 2020).

Additional E3 ligases other than Parkin –such as Mitochondrial ubiquitin ligase 1 (MUL1) – can ubiquitinate mitochondrial membrane proteins and inhibit the mutant phenotypes of Parkin in the ubiquitin-mediated pathway (Yun et al., 2014). Knocking out MUL1 with either PINK or Parkin at the same time had more serious phenotypes than individual nulls. These data suggest that MUL1 may be an alternative pathway, its function is parallel to Parkin partially. Both mitochondrial autophagy mediated by Parkin and MUL1 are dependent on PINK and both regulate the activity of Mfns (Gegg et al., 2010). Other E3 ligases like G78, which aim the common targets with Parkin, whether these E3 ligases mediated mitochondrial autophagy is dependent on PINK1 needs to be further investigated (Fu et al., 2013). Thus, in the Parkin mutation model, compensation by ubiquitin-mediated mitophagy may be a potential repair mechanism for the balance of mitophagy.

**FIGURE 2:**
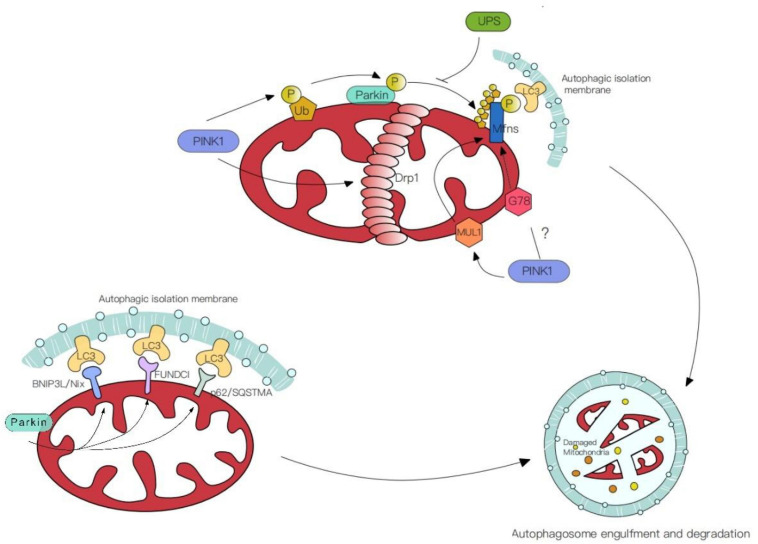
Mechanisms of mitophagy. (1) PINK/Parkin mediated mitophagy. PINK1 phosphorylates the ubiquitin molecules (Ub) on the mitochondrial surface or straightforwardly phosphorylate ubiquitin-like domain (Ubl) of Parkin. Simultaneously, PINK1 facilitates the aggregation of Drp1 in OMM, leading to mitochondrial fragmentation and thus promoting mitophagy. (2) Ubiquitin-mediated mitophagy. E3 ligases like Mitochondrial ubiquitin ligase 1 (MUL1) and G78 ubiquitinate the common targets with Parkin. (3) Receptor-mediated mitophagy. Autophagic receptors (BNIP3L/Nix, p62/SQSTMA, FUNDCI) ubiquitinated by Parkin on the OMM, contact with MAP1LC3/LC3-interacting regions (LIRs) and mediate the damaged mitochondria degradation.

Mitochondrial molecular mechanisms of the PINK1-independent pathway have been revealed by studies in human cell lines and animal models (Goldberg et al., 2003; Perez and Palmiter, 2005; Kitada et al., 2007; Akundi et al., 2011). On the OMM, proteins ubiquitinated by Parkin can directly interact with the autophagic isolation membrane via autophagic receptors including BNIP3L/Nix, p62/SQSTMA, FUNDCI (Figure 2). This receptor-mediated mitophagy utilizes autophagic receptors with MAP1LC3/LC3-interacting regions (LIRs), these receptors gather and mediate the damaged mitochondria degradation with the help of LIRs motifs. The BHRF1 protein (a BCL2 homolog) is one of the autophagic receptors that can induce mitochondrial fission and degradation, thus resisting innate immunity activation (Vilmen et al., 2020). However, another study shows the damaging role of BHRF1 within hypoxia brain and spinal cord injury (Shi et al., 2014; Yu et al., 2018). Unraveling these specific roles further could facilitate and identify possible therapeutic targets.

In addition to receptor-mediated or ubiquitin-mediated mitophagy, the roles of non-selective mitochondrial clearance, the endosome-lysosome pathway, and mitochondrial derived vesicles (MDV) in mitochondrial quality control processes have attracted the attention of researchers. The latter two maintain mitochondrial health by transporting impaired mitochondria to lysosomes for degradation or selective clearance of the impaired parts of mitochondria (Hammerling et al., 2017). These pathways are also essential for the maintenance of synaptic mitochondrial health. Yet, future work should elucidate the mechanisms involved in mitochondrial quality control within the distal axon.

## Mitochondrial Transport

Mitochondria serve as local energy sources and maintain various essential functions at synaptic terminals. Lack of synaptic mitochondria is a common feature of some instances of age-related neurodegeneration. Because neuronal synapses require healthy mitochondria to maintain their functions, the transport and degradation of damaged mitochondria and the correct distribution of healthy mitochondria are essential processes.

The mechanism underlying mitochondrial maintenance at synaptic terminals is poorly understood. Newly synthesized organelles should transport along the axon to supplement mitochondria at the remote end of the axon (Sheng, 2014; Zheng et al., 2019). In previous research, cells depleted for Drp1 resulted in mitochondrial elongation due to organelle division deficiency and impaired mitophagy, which then form large spheres. This abnormal form of mitochondria may be difficult to transport into the long dendritic and axonal extensions (Kageyama et al., 2012). Interestingly, fusion and fission can also facilitate the isolation of dysfunctional mitochondria. For example, through fusion and fission events, mitochondria with damaged or healthy components merge into a new organelle, following that, it undergoes fission to distribute components evenly (Youle and van der Bliek, 2012).

**FIGURE 3:**
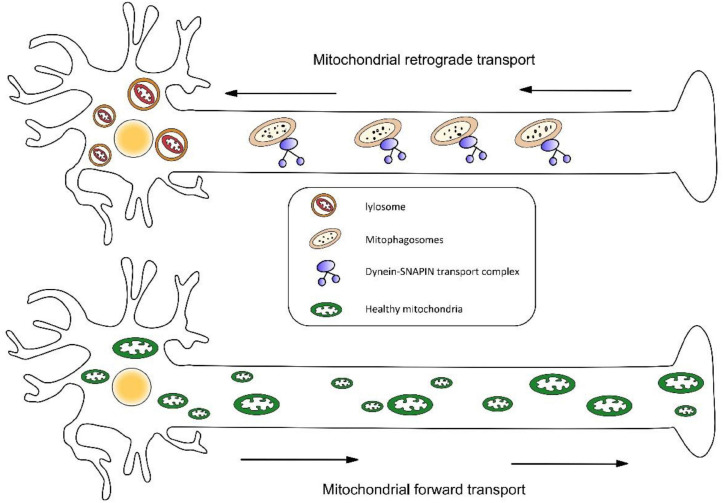
(1) Mitophagosomes fuse with dynein-SNAPIN transport complex-loaded LEs and move exclusively in a retrograde direction in axons. Next, lysosomes located in the soma of neurons degrade damaged mitochondria. (2) Newly synthesized organelles should transport along the axon to supplement mitochondria at the remote end of the axon.

Similarly, retrograde transport may also be essential to mitochondrial clearance and repair. The latest research shows mitophagy coordination with retrograde transport of mitochondria (Cai et al., 2012). Three proteins were highlighted in this study—RHEB (Ras homolog enriched in brain), BNIP3L/Nix (an outer mitochondrial membrane protein), and dynein-SNAPIN motor-adaptor complex. RHEB was previously reported to target mitochondria for autophagy in non-neuronal cells. This study determined that RHEB-targeted mitochondria exhibit high motility and display dominant retrograde movement in neuronal axons. It also mentions that membrane protein BNIP3L/Nix facilitates RHEB association with damaged mitochondria, then these combinations are engulfed by phagophores to form mitophagosomes, which move exclusively in a retrograde direction in axons (Figure 3). It is known that degradative lysosomes primarily are located in the soma of neurons for mitophagic clearance. Increasing SNAPIN levels in AD neurons decreases mitophagic retention and attenuates mitochondria defects in axons by enhancing retrograde transport motility of mito-amphisomes (Mitophagosomes fuse with dynein-SNAPIN transport complex-loaded LEs). This study provides new mechanistic insights into how mitophagy coordinates with retrograde transport of synaptic mitochondria and how the failure of retrograde transport aggravates AD-associated mitochondrial defects and synaptic degeneration.

The bidirectional movement of axonal mitochondria via long-distance transportation is regulated by mitochondrial Rho (Miro) and trafficking kinesin-binding protein (TRAK)1 and 2 (Fransson et al., 2006; Wang and Schwarz, 2009; van Spronsen et al., 2013). Interestingly, Mfn2 has been shown to directly regulate axonal mitochondrial transport, by interacting with Miro and complex that links mitochondria to kinesin motors (Misko et al., 2010). Miro has been linked to three proteins– PINK1, Parkin, and LRRK2, which all have been reported to promote Miro degradation and mitophagy (Wang et al., 2011; Liu et al., 2012; Hsieh et al., 2016). Mutations in LRRK2 are the commonest pathogenesis of PD (Corti et al., 2011), in this case, Miro remains on damaged mitochondria thus hindering mitochondrial degradation. This section on the pathogenesis of PD will be discussed below.

## Alzheimer’s Disease

Alzheimer’s disease (AD) is characterized by the progressive loss of cholinergic neurons in brain regions that are critical for memory, language, and learning, resulting in early memory loss, which can even progress to broad cognitive impairment. Two major hallmarks of AD are hyperphosphorylated tau and amyloid beta (Aβ) plaques in Neurofibrillary tangle (Bird, 1993). By the role of BACE-1 (known as β-secretase) and subsequent cleavage of the complex, small peptide Aβ is secreted from membrane-bound amyloid precursor protein (APP) to the extracellular fluid. These Aβ products are oligomerized, then mature into fibrous structures and localize in neuronal tissues as amyloid plaques, which are thought to be neurotoxic. Over-expression of APP causes an imbalance of mitochondrial fission/fusion that contributes to mitochondrial excessive fragmentation and abnormal distribution accumulating around the perinuclear area (Wang et al., 2008). Tau protein is a microtubule-associated protein (MAP), the phosphorylation of tau occurs step by step, and hyperphosphorylated tau proteins have the most impact on the memory function (Johnson and Stoothoff, 2004; Shahpasand et al., 2012; Ma et al., 2017; Abtahi et al., 2020). Hyperphosphorylated tau causes microtubule instability, which leads to neuronal synaptic disruption, connectivity and plasticity damage, neuronal death ultimately. A study has shown that caspase-cleaved tau increases TRAK2-mitochondria binding and decreases the ATP production available for the locomotion of these organelles, resulting in adverse effects on mitochondrial transport (Quintanilla et al., 2020).

Impairment of Mitochondrial quality control is an early salient feature in susceptible neurons of an AD patient brain, characterized by distinct mitochondrial fragmentation, abnormal mitochondrial distribution, and mitochondrial dysfunction (Wang et al., 2017), culminating in neurodegeneration. Although both tau and Aβ play a role in oxidative stress and mitochondrial damage, it still seems difficult to distinguish which of them is more closely related to cognitive impairment and neurodegeneration in AD. However, a pathological promoting effect between these two characteristic changes has been demonstrated. Aβ plaques provide a favorable environment for the aggregation of Alzheimer’s brain tau-seeded, which was followed by the formation of neuropil threads (NTs) (He et al., 2018). Aβ plaques, pathologic tau, and a-synuclein were found to co-exist in the brains of neurodegenerative diseases patients with dementia. It was shown that α-synuclein bridges the interaction between Aβ plaques and tau tangles. By accelerating the diffusion of α-synuclein, Aβ plaques induce hyperphosphorylation of tau and ultimately exacerbate neuronal damage and neuronal damage (Bassil et al., 2020). Using immunofluorescence techniques, studies have also observed a selective contact between Drp1 and phosphorylated tau, which facilitates the interaction between Drp1 and Aβ, resulting in excessive mitochondrial fragmentation. The pathological synergy between Aβ plaques and hyperphosphorylated tau has been observed but the underlying mechanisms require further investigation.

In the early stage of AD, studies have revealed that there is a greater increase in Drp1 compared to Marf (homologous to human MFN2) expression. However, with disease progression, both Mfn2 and Mfn1 levels are downregulated markedly in the brain of AD patients (Manczak et al., 2011). A reduction in the expression of fusion genes is thus likely to be a later event in AD and is associated with the progress of the AD process. Although changes in protein rates have been observed, the triggers remain poorly understood, and whether the process of mitochondrial fragmentation is caused by this GTPase change, and it is positively correlated with the progression of AD cases is a question that merits further investigation. The feasibility of mitochondrial dynamics as a treatment target for neurodegeneration requires further supporting studies.

The expression of PINK1 was markedly lower in the brains of patients with AD or APP transgenic mice than controls (Du et al., 2017). AD models undergo defective mitophagy that results in abnormal mitochondria accumulation and promotes Aβ production, while Aβ accumulation may exacerbate impaired mitophagy (Kerr et al., 2017; Fang, 2019; Kingwell, 2019). Valinomycin activates mitophagy via the PINK/Parkin signaling pathway. N2a/APP695swe cells treated with valinomycin for 3h can recover PINK1/Parkin-mediated mitophagy and the removal of damaged mitochondria thus reduces Aβ and restores ATP levels. Notably, the different action times of valinomycin may produce opposite results (Xiong et al., 2020). β-asarone has also been shown to improve learning and memory in Aβ1-42-induced AD rats by regulating PINK1-Parkin-mediated mitophagy (Han Y. et al., 2020). Studies have demonstrated a vicious cycle formed by the interaction between mitochondrial dysfunction, phosphorylated-Tau, and impaired mitophagy (Kerr et al., 2017). The mitochondrial division inhibitor 1 (mdivi-1) has been reported as an inhibitor of dynamin related protein 1 (Drp1) (Wang et al., 2017), mdivi-1 or the knockdown of Drp1 downregulates mitochondrial ROS levels and blocks LPS-induced cellular inflammatory activation. However, a recent study reported that the neuroprotective effect of mvidi-1 is mainly via the regulation of mitochondrial function and intracellular calcium signaling, rather than relying on Drp1 (Ruiz et al., 2018). The role of mvidi-1 in inhibiting mitophagy pathway impairment in a traumatic brain injury model has also been proposed (Wu et al., 2018).

Accumulating experimental data suggest that the impairment of the PINK/Parkin-dependent or -independent mitophagy pathway is critical in AD pathology. Here, some outstanding questions deserve further exploration—Is defective mitophagy in AD an early preceding event and cause of the subsequent accumulation of toxic proteins? What are other possible molecular mechanisms of defective mitophagy in AD?

Neuroinflammation is a common pathological feature of neurodegeneration. Immune training induced by peripheral inflammatory stimuli exacerbates cerebral β-amyloidosis (Wendeln et al., 2018). Mitophagy attenuates inflammation by limiting the secretion of inflammatory cytokines and regulating the homeostasis of immune cells (Xu et al., 2020), impairment of mitochondrial quality control has been proven to contribute to the activation of innate immune pathways (Guerriero et al., 2017). In addition, oxidative stress can be triggered by either Aβ or pathological tau. This level is in proportion to the rate of tau and Aβ aggregations (DuBoff et al., 2012; Kandimalla et al., 2016; Manczak et al., 2016). Previous studies have shown that oxidative stress can perturb the mitochondrial fission/fusion balance (Wang and Chen, 2016; Jezek et al., 2018), thus, the interaction between impairment of mitochondrial quality control, pathological tau or Aβ, and oxidative stress may also form a vicious circle.

In addition to the brain, peripheral tissues can also generate Aβ, and the hypothesis that Alzheimer’s disease is a systemic disease has been proposed. Using a model of parabiosis between AD mice and controls, researchers observed that Aβ entered the brains of wild mice and subsequently formed Aβ plaques (Bu et al., 2018). Clinical studies have also explored the feasibility of using peripheral blood Aβ to predict intracerebral amyloid-beta-positive or -negative status (Nakamura et al., 2018). Significant changes in the expression levels of Fission1 (FIS1), Drp1, and Parkin were observed in peripheral blood samples from AD patients, these gene expressions correlate with cognitive performance (Pakpian et al., 2020). Thus, the genetic profiling of peripheral blood mitochondrial dynamics in AD patients may act as an underlying predictor of mitochondrial function in the brain and a potential consideration for blood AD biomarkers.

## Parkinson’s Disease

Parkinson’s Disease (PD is the second most common neurodegenerative disease, caused by environmental factors that interact with genetic susceptibility. It is characterized by the progressive loss or degeneration of the dopaminergic (DA) neurons in the substantia nigra and the accumulation of a-synuclein in DA neurons, characterized by a deficit in a patient’s ability to move (Alexander, 2004). Mutant α-synuclein mislocalizes to mitochondria, it reacts with spectrin to prompt Drp1 translocation (Ordonez et al., 2018), resulting in mitochondrial fragmentation. Lewy body is an intracerebral marker of PD patients, the misfolded a-synuclein enters to Lewy body and aggregates abnormally (Dickson et al., 2008). In addition, aging, oxidative stress, and traumatic brain injury also play roles in increasing the risk of Parkinson’s disease. Methyl-4-phenylpyridinium (MPP +) is the active metabolite of 1-methyl-4-phenyl-1,2,3,6-tetrahydropyridine (MPTP), it enters nigrostriatal dopamine neurons selectively, causing extensive impairment of the respiratory chain to promote oxidative stress (Zhang et al., 2010). There has been reported that MPP + induces excessive mitochondrial fission by engendering S-nitrosylation of Parkin and triggering the phosphorylation of Drp1 Ser616 (Zhang et al., 2016).

Fly models of PINK1 and Parkin mutations summarize the dominant hallmarks of PD, including mitochondrial dysfunction, motor impairment, loss of dopaminergic neurons, and shortened lifespan (Yang et al., 2006), impairment of the PINK/Parkin pathway points to a possibility of an overlapping effect between PD and AD (Sliter et al., 2018; Xu et al., 2020). Mitophagy has been thought to counteract neuronal damage through mitochondrial quality control, while its role in anti-neuroinflammation is unclear. A study addressing the role of mitophagy in innate immunity suggests that PINK and Parkin may be a category of inflammatory suppressors and that PINK-Parkin-mediated mitophagy can restrain innate immunity and alleviate the inflammatory phenotype (Rakovic et al., 2019). Interestingly, Parkin activated by activated protein kinase (AMPK) may act as a tumor suppressor. By promoting polyubiquitination of RIPK3 (a serine/threonine-protein kinases), the AMPM-Parkin axis blocks the formation of necrosome RIPK1-RIPK3 and inhibits inflammation-associated tumorigenesis (Lee et al., 2019). Research on Parkin has established that PINK1/Parkin-mediated mitophagy links to PD pathogenesis at least in part via mitochondrial quality control (Rakovic et al., 2019). Thus, combating PD by exploring a molecular diagnostic based on the activity of Parkin and other treatments directed at Parkin-mediated mitophagy may be an effective strategy.

A critical mechanism by which Parkin activation is regulated is ubiquitination, which is dependent on the ubiquitination of ubiquitinating enzymes. De-ubiquitination is mediated by de-ubiquitinating enzymes (DUBs), it is the reverse of ubiquitination and in charge of reversing mono- or polyubiquitination in proteins. Almost 100 DUBs have been identified in human genes, and the ubiquitin-specific protease (USP) family is the largest branch of them. The general theory is that DUBS affects mitophagy by regulating: (i) activity of Parkin, (ii) stability of Parkin, (iii) proteasome activity, and autophagy. Under normal conditions, a-synuclein is ubiquitinated and circulates in the brain; USP13 may reverse the ubiquitination of a-synuclein and exacerbate the process of neurodegeneration (Liu et al., 2019). USP15 may inhibit Parkin-mediated ubiquitination of mitochondrial surface proteins rather than reversing the ubiquitinated state of Parkin to impede mitophagy (Cornelissen et al., 2014). Thus, the knockdown of USP family members or USP inhibition may be an effective way to ameliorate mitochondrial deficiency. In addition to PINK and Parkin, mutations and/or overexpression in the majority of proteins involved in familial forms of PD, including LRRK2, DJ-1, and VPS35, have been proven to impair mitochondrial quality control and neuroprotective effects (West et al., 2005). LRRK2 mutations encode leucine-rich repeat kinase 2 (LRRK2), which is the commonest cause of PD and is associated with an increase in kinase activity (Wang et al., 2012) interacting with Drp1 to cause mitochondrial fragmentation (Yue et al., 2015; Bonello et al., 2019). Recent studies show that LRRK2 interferes with the interaction between Parkin and Drp1, while suppression of LRRK2 kinase activity attenuates this interaction (Bonello et al., 2019). As mentioned above, Miro retention on injured mitochondria in the LRRK2 mutant delays effective degradation and disturbs mitochondrial transport (Wang et al., 2016). Disruption of either the forward or retrograde mitochondrial transport system or production of defective mitochondria results in a decreased proportion of healthy mitochondria and increased accumulation of damaged mitochondria at synapses. Furthermore, LRRK2 was still recruited to mitochondria in PD fibroblast lines expressing mutant PINK1 and Parkin, indicating that LRRK2 function may be a comparable pathway to PINK/Parkin. More studies are needed to elucidate the mechanisms by which LRRK2 regulates mitophagy and mitochondrial phagocytosis.

Mutations in VPS35 play a key role in the retromer complex. It has been reported that mutant VPS35 increases the clearance of inactive Drp1 complexes, which leads to mitochondrial fragmentation (Ordonez et al., 2018). Another study observed that VPS35 aids in the removal of MUL1 from the OMM, thus inhibiting mitochondrial fusion by preventing Mfn2 ubiquitination (Tang et al., 2015). Similar to VPS35 mutation, the DJ-1 protein is one of the rare causes of PD. It is linked to pathogenic conditions of PD, but the basic molecular mechanisms remain poorly understood. Flies deleted for DJ-1 manifest a similar pathological phenotype as PINK1/Parkin mutants. DJ-1 knockdown alleviates PINK mutation, but not Parkin, indicating that DJ-1 may act in parallel to PINK/Parkin. However, this hypothesis has not been conclusively tested.

## Huntington’s Disease

Huntington’s disease (HD) is a dominantly inherited neurodegenerative disease caused by mutations that result in a duplication of the CAG triplet in the polyglutamine (polyQ) region of the huntingtin protein (Htt) (Ghosh and Tabrizi, 2018). HD is characterized by the progressive loss of the capacity to control movements, cognition, and emotional expression and with a wide spectrum of other signs. Neurodegeneration mainly occurs in the striatum and cerebral cortex. Mitochondrial fragmentation in the peripheral blood cells of HD patients has been described. They show the level of Mfns and Opa1 decreased as the amount of Drp1 increased. A new study shows that the use of the P110 (an inhibitor of Drp1-Fis1 interaction) to inhibit Drp1 hyperactivation is adequate to mitigate HD-associated behavioral impairments and neuropathology (Zhao et al., 2018). This may help develop a potential therapeutic agent for treating HD. A previous study established that the mutant Htt can enhance the GTPase activity of Drp1 by interacting with it on mitochondria.

Another study has suggested a possible mechanism of this interaction, they discovered that mutant Htt may contribute to the overproduction of NO. Drp1 reacts with NO, leading to S-nitrosylation of Drp1, leading to excessive mitochondrial fission and neuronal injury. Remarkably, this SNO-Drp1 has also been observed in AD. In addition, this study also shows that mutant Htt can also be S-nitrosylated, so that it may transnitrosylate or transfer NO to Drp1, leading to S-nitrosylation of Drp1. The study also suggested that S-nitrosylation of Drp1 could facilitate the interaction between Drp1and the mutant Htt (Song et al., 2011; Sesaki et al., 2014). Consistently, introducing the non-nitrosylatable mutant Drp1 (C644A) ameliorated the adverse effects of abnormal mitochondrial dynamics on neurons caused by mutant Htt. These studies suggest that there is an association between the pathological progression of HD and S-nitrosylation of Drp1.

Mutant Htt directly impairs the potential of the mitochondrial membrane, and calcium homeostasis. Increased levels of cytoplasmic calcium could activate Drp1 through dephosphorylation by the calcium-dependent protein phosphatase calcineurin, and promote its association with mitochondria (Costa et al., 2010). Moreover, Mitogen-activated protein kinase 1 (MAPK1) can interact with Drp1 and phosphorylates it at Ser-616 causing the overactivation of the Drp1 in HD knock in mouse-derived striatal cells (Qi et al., 2019).

Htt has been proposed as a scaffold protein with multiple protein-protein interaction regions participation in axonal vesicle transport, autophagosome, and mitochondria (Gauthier et al., 2004; Trushina et al., 2004; Wong and Holzbaur, 2014). Recently, the role of Htt as a scaffold protein for the launch of stress-induced selective autophagy has emerged. One study shows that Htt interacts with ULK1 (unc-51 like kinase 1) and SQSTM1/p62 (a selective macro-autophagy receptor), which bind simultaneously to autophagosomes by acting with LC3-II (Ochaba et al., 2014; Rui et al., 2015). Htt-depleted neurons or polyQ-Htt-expressing neurons display inefficient degradation of engulfed mitochondrial fragments (Franco-Iborra et al., 2020). Impairment of the selective degradation of mitochondria by autophagy causes mitochondrial dysfunction and the accumulation of damaged mitochondria and consequently contributes to neurodegeneration. In addition, the polyQ tract in mHtt affects the interaction of LC3-II with mitophagy receptors including OPTN and CALCOCO2, which plays an irreplaceable role compared to other receptors (Lazarou et al., 2015). Htt functions as a scaffolding protein, aggregating the different proteins required for mitophagy to take place, and these interactions can be altered by Htt with polyQ and the stability of the protein complex can also be destabilized by it. For example, abnormal interaction between GAPDH and polyQ stalled the GAPDH-induced mitophagy, and, GAPDH fails to induce direct engulfment of damaged mitochondria into lysosome (Hwang et al., 2015).

## Conclusion and Future Perspectives

Abnormalities of mitochondrial dynamics seem to be a common feature of many neurodegenerative diseases. Changes in the number and GTPase activity of Drp1 frequently appear in the early pathological process of neurodegenerative disease, accompanied by excessive mitochondrial division, mitochondrial transport damage, and abnormal mitochondria mitophagy (Corti et al., 2020; Pradeepkiran and Reddy, 2020), etc. Various related experiments better help us understand that the changes in mitochondrial dynamics may be different in different neurodegenerative diseases, and even different stages of the same disease. Research on mitochondrial dynamics can help us restore mitochondrial function in a targeted manner. For example, a large number of experiments have proved that introducing dominant-negative Drp1 K38A mutant or inhibiting the binding of Drp1 to its mitochondrial receptor can effectively inhibit the excessive division of mitochondria. On another hand, the latest research directions lean toward the mitochondrial autophagy pathway, and treatment options for mitochondrial autophagy are constantly evolving (Fang et al., 2019; Zhao et al., 2020).

In this review, we discussed the interaction between three types of mitochondrial dynamics and three typical neurodegenerative diseases. We also discussed the internal interactions of mitochondrial dynamics, and it seems to point in one direction—mitochondrial quality control. Several proteins have been frequently mentioned in previous studies, such as Drp1 and PINK, which have been shown to influence mitochondrial dynamics through multiple mechanisms. At the same time, with the study of new mitophagy pathways, a growing number of researchers are starting to notice the effects of other pathways on mitochondrial dynamics. This may bring forth new targets for alleviating, and perhaps even curing, neurodegenerative diseases.

The role of astrocytes has been shown during the mitophagic degradation of damaged mitochondria from adjacent neurons (Davis et al., 2014). In the latest research, researchers have found that mitochondrial fragments can also be transmitted between microglia, astrocytes, and neurons to spread inflammatory neurodegeneration, which contributes to the production of neurodegenerative diseases such as AD, PD, HD (Joshi et al., 2019). The results of this study may be related to the previously observed increased glial fibrillary acidic protein (GFAP) in AD, and support the hypothesis that the activation of astrocytes and microglia will induce neurodegeneration. However, the molecular mechanism of this course requires additional experimental studies. As the majority of previous research efforts has focused more on the ubiquitination of astrocytic GFAP and microglia or the S-nitrosylation of GFAP, new exploratory studies provide us with valuable ideas for better understanding the relationship between mitochondrial dynamics and neurodegeneration.

Previous studies of mitochondrial dynamics types have focused on synaptic mitochondrial transport. Mitochondrial transport between astrocytes and microglia shows that mitochondrial fragments can not only cause mitochondrial quality control disorders at neuronal synapses but also that they can be transmitted as a “diffusible signal,” which contributes to the occurrence of neurodegenerative diseases. This suggests that mitochondrial dynamics are linked with cell fate (Iwata et al., 2020).

In conclusion, although we cannot completely cure neurodegenerative diseases at the current level of technology, previous studies have demonstrated that mitochondrial dynamic abnormalities and dysfunction can affect the pathogenesis of neurodegenerative diseases in many ways. More importantly, there are complex and inseparable relationships between various types of mitochondrial dynamics disorders. Therefore, it is particularly important to clarify the cause-and-effect relationships between them, pointing us to future directions for the treatment of neurodegenerative diseases.

## Author Contributions

The manuscript has not been published previously, in whole or in part, and it is not under consideration by any other journal. All authors are aware of and accept responsibility for the manuscript.

## Conflict of Interest

The authors declare that the research was conducted in the absence of any commercial or financial relationships that could be construed as a potential conflict of interest.
